# Dectin-1 Is Essential for Reverse Transcytosis of Glycosylated SIgA-Antigen Complexes by Intestinal M Cells

**DOI:** 10.1371/journal.pbio.1001658

**Published:** 2013-09-17

**Authors:** Nicolas Rochereau, Daniel Drocourt, Eric Perouzel, Vincent Pavot, Pierre Redelinghuys, Gordon D. Brown, Gerard Tiraby, Xavier Roblin, Bernard Verrier, Christian Genin, Blaise Corthésy, Stéphane Paul

**Affiliations:** 1 GIMAP/EA3064, INSERM CIE3 Vaccinology, Université de Lyon, Saint-Etienne, France; 2 Cayla-InvivoGen, Toulouse, France; 3 Institut de Biologie et Chimie des Protéines, FRE3310/CNRS, Université de Lyon, France; 4 Section of Infection and Immunity, Institute of Medical Sciences, University of Aberdeen, Aberdeen, United Kingdom; 5 R&D Laboratory of the Division of Immunology and Allergy, Centre Hospitalier Universitaire Vaudois, Lausanne, Switzerland; Scripps Research Institute, United States of America

## Abstract

Intestinal microfold (M) cells possess a high transcytosis capacity and are able to transport a broad range of materials including particulate antigens, soluble macromolecules, and pathogens from the intestinal lumen to inductive sites of the mucosal immune system. M cells are also the primary pathway for delivery of secretory IgA (SIgA) to the gut-associated lymphoid tissue. However, although the consequences of SIgA uptake by M cells are now well known and described, the mechanisms whereby SIgA is selectively bound and taken up remain poorly understood. Here we first demonstrate that both the Cα1 region and glycosylation, more particularly sialic acid residues, are involved in M cell–mediated reverse transcytosis. Second, we found that SIgA is taken up by M cells via the Dectin-1 receptor, with the possible involvement of Siglec-5 acting as a co-receptor. Third, we establish that transcytosed SIgA is taken up by mucosal CX3CR1^+^ dendritic cells (DCs) via the DC-SIGN receptor. Fourth, we show that mucosal and systemic antibody responses against the HIV p24-SIgA complexes administered orally is strictly dependent on the expression of Dectin-1. Having deciphered the mechanisms leading to specific targeting of SIgA-based Ag complexes paves the way to the use of such a vehicle for mucosal vaccination against various infectious diseases.

## Abstract 2

This work reports the long-awaited identification of Dectin-1 and Siglec-5 as the M cell co-receptors that mediate the reverse transcytosis of secretory IgA molecules to mount a gut immune response.

## Author Summary

Secretory IgA (SIgA) antibodies are secreted into the gut lumen and are considered to be a first line of defense in protecting the intestinal epithelium from gut pathogens. SIgA patrol the mucus and are usually known to help immune tolerance via entrapping dietary antigens and microorganisms and other mechanisms. SIgA, in complex with its antigens, can also be taken back up by the intestinal epithelium in a process known as reverse transcytosis. SIgA can thereby promote the uptake and delivery of antigens from the intestinal lumen to the Gut-Associated Lymphoid Tissues (GALT), influencing inflammatory responses. This reverse transcytosis of SIgA is mediated by specialized epithelial M cells. Because M cells possess the ability to take up antigens and are therefore important to the local immune system, they are a key target for the specific delivery of novel mucosal vaccines against various diseases. M cell receptors that take up the SIgA-antigen complexes, which serve as mucosal vaccine vehicles, represent an important aspect of this vaccine strategy. The identification of SIgA receptor(s) on the surface of M cells has, however, remained elusive for more than a decade. In this study, we now identify Dectin-1 and Siglec-5 as the key receptors for M cell–mediated reverse transcytosis of SIgA complexes. We further find that the glycosylation modification, and particularly sialylation, of SIgA is required for its uptake by M cells. We show that, when administered orally in complex with SIgA, the HIV p24 antigen is taken up in a strictly Dectin-1-dependent manner to stimulate a mucosal and systemic antibody response. These findings are considered important for understanding gut immunity.

## Introduction

The mucosal immune system comprises the largest part of the entire immune system, and the mucosal surface represents the primary site of entry for pathogenic agents. SIgA has long been recognized as a first line of defense in protecting the intestinal epithelium from enteric pathogens and toxins. It is generally assumed that SIgA acts primarily through receptor blockade, steric hindrance, and/or immune exclusion. In recent years evidence has emerged indicating that SIgA promotes the uptake and delivery of Ags from the intestinal lumen to DC subsets located in gut-associated lymphoid tissues (GALTs), and influences inflammatory responses normally associated with the uptake of highly pathogenic bacteria and potentially allergenic antigens. This particular feature of SIgA, called reverse transcytosis, is mediated by epithelial M cells [1]. However, although the potentially useful properties of M cells on SIgA uptake are now well known, the receptor(s) whereby SIgA is taken up and transported by M cells remain(s) elusive.

SIgA reverse transcytosis was first invoked to account for the binding of rabbit SIgA to M cells in Peyer's patches (PPs) of suckling rabbits [2]. Colloidal gold particles coated with IgA were subsequently detected within M cell cytoplasmic vesicles and in the extracellular space of M cell pockets [3]. Endogenous SIgA was also shown to bind to human PP M cells in paraffin sections of human ileum [4]. In frozen sections, labeled SIgA could be visualized bound at the apical surface, in transit through intracellular vesicles, in the intraepithelial pocket, and on basolateral processes extending toward the basal lamina. In a mouse ligated ileal loop assay, mouse SIgA, human SIgA2, but not human SIgA1, bound to PP M cells [4]. Structural changes could explain the differences in reverse transcytosis between these subtypes. The IgA1 hinge features a 16 amino-acid insertion, lacking in IgA2, comprising a repeat of eight amino acids decorated with 3–5 O-linked oligosaccharides [5],[6]. Recombinant IgA1 with a deleted hinge region gained M cell binding function, which was interpreted as the M cell's binding site comprising both domains Cα1 and Cα2, juxtaposed in mouse IgA and human IgA2 [4]. Overall, IgA2 contains 4 N-glycosylation sites (Asn^166^, Asn^263^, Asn^337^, Asn^459^). In dimeric IgA, the Fc regions of the two monomers are linked end to end through disulfide bridges to the J chain [7]. IgA, with or without bound secretory component (SC), selectively adheres to the apical surfaces of mouse PP M cells [4].

To date, only a limited number of M cell receptors and their ligands have been identified, but most of these receptors are expressed in M cells and neighboring enterocytes as well. Some important pathogen recognition receptors, such as toll-like receptor-4, platelet-activating factor receptor, and α5β1 integrin have been identified on the surface of human and mouse M cells [8],[9]. The sialyl Lewis A (CA19.9) antigen lectin reacts with 80% of human M cells and, in contrast to the other ligands, binds only weakly to the enterocytes of the follicle-associated epithelium (FAE). Moreover, there is a wide variation in marker expression between M cells of different species and even between M cells at different portions of the intestine within the same species [10]. Indeed, M cells in murine, but not human, PP are preferentially bound with Ulex europaeus agglutinin–1 (UEA-1), a lectin specific to α-l-fucose residues [11]. A first mouse M cell–specific monoclonal antibody (mAb NKM 16-2-4) [12] displaying specificity for α(1,2)-fucose–containing carbohydrate moieties was produced. Glycoprotein 2 (GP2) was also shown to be specifically expressed on M cells of mouse and human PPs [13]–[15] and serves as an endocytic receptor for luminal antigens [16]. Another M cell marker, clusterin, is expressed in M cells and follicular DCs at inductive sites of human GALTs [14].

To date, the molecular partner(s) involved in SIgA reverse transcytosis has(have) not been identified in mice or in humans. In this work, we sought to map the structural feature(s) responsible for the selective interaction between murine SIgA and M cells. Since it is impossible to keep M cells in culture, one valuable approach consists in using cell culture models that mimic essential features of the FAE tissue. An *in vitro* model was used, based on the co-culture of polarized Caco-2 cells grown on inverted inserts and exposed to human Raji B lymphocytes [17],[18]. Following optimization in terms of functionality and reproducibility, we evaluated the transport of wild-type (wt) and mutant human IgA2 across newly differentiated M-like cells in comparison with other Ab isotypes. We found that glycosylation sites and in particular sialylation of the Cα1 region of IgA2 are required for M-like cell-mediated reverse transcytosis. We demonstrate for the first time that Dectin-1 expressed on the surface of M cells acts as a receptor involved in SIgA reverse transcytosis both *in vitro* and *in vivo*. Siglec-5 receptor seems also to participate in reverse transcytosis. Such a selective interaction has functional consequences *in vivo*, since targeting of HIV p24-SIgA complexes after oral delivery promotes the production of systemic and mucosal Ag-specific Abs in wt mice only, and not in Dectin-1 KO animals.

## Results

### Establishment of an *in Vitro* Model of Human FAE

**Figure 1:**
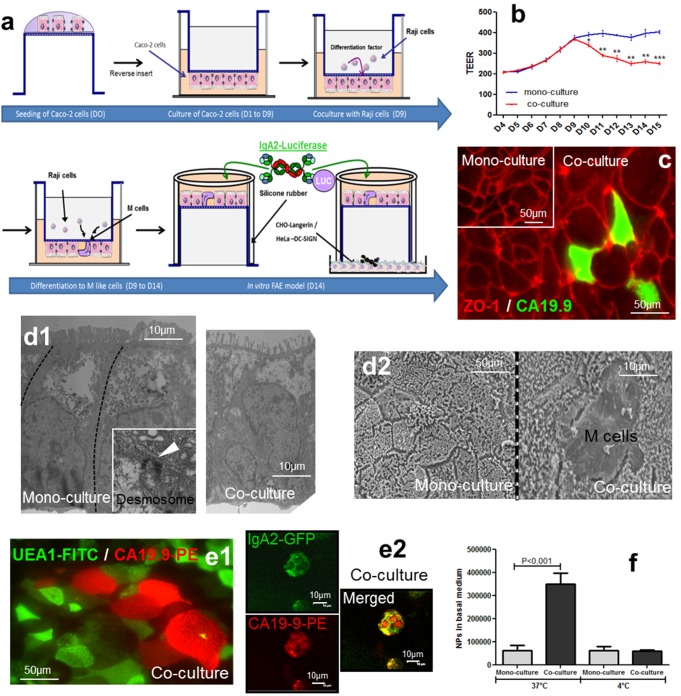
Validation and characterization of the inverted *in vitro* model of human FAE. (a) Schematic representation of the successive steps leading to the establishment of the M-like cell-containing Caco-2 cell monolayer. Molecular and cellular partners added as a function of the experimental setting are depicted. D, day. (b) Slight decrease of transepithelial electrical resistance (TEER) was observed during M cell conversion of polarized Caco-2 cell monolayer. (c) Localization by immunofluorescence of ZO-1 and CA19.9 in the mono- and co-culture models shows tight intercellular junctions around CA19.9^+^ M cells (top view). (d1) Identification of M-like cells by transmission electron microscopy (TEM). In co-culture, M-like cells were identified by the effacement of microvilli at the apical surface and the presence of enfolded lymphocytes (side view). Mono-cultures present a columnar shape as well as a brush border. (d2) Scanning electron microscopy (SEM) confirmed the presence of M-like cells and the lack of microvilli at their apical surface (top view). (e1) Identification and localization of M-like cells and enterocyte cells by immunofluorescence staining with anti-CA19.9-PE mAb and lectin UEA-1-FITC, respectively (top view). (e2) Co-localization evaluated by immunofluorescence microscopy of CA19.9-PE and IgA2-GFP indicates binding of the Ab to M-like cells (top view). (f) Increased apical-to-basolateral transport of 0.2 µm yellow-green-conjugated NPs across the co-cultures as compared to mono-cultures. Absence of transport at 4°C is indicative of an active membrane trafficking process. Each set of experiments was repeated at least twice.

The model was adapted as described in the Methods section to optimize its reproducibility (Figure 1a). Prior to adding the lymphocytes, the tightness of the Caco-2 cell monolayer was checked by measuring transepithelial electrical resistance (TEER). The decrease in TEER observed after 5 d of co-culture is indicative of Caco-2 cell conversion into M cells (Figure 1b) [19] but not a result of the deterioration of tight junction organization, as reflected by preserved ZO-1 immunolabeling (Figure 1c), of either mono- or co-cultures.

M cells display a reduced brush border at their apical surface and an invaginated basolateral membrane, forming a pocket filled with immunoreactive cells [3]. Transmission electron microscopy shows that mono-cultures of Caco-2 cells exhibit a well-developed brush border with tightly packed microvilli, whereas in co-cultures with Raji cells, M-like cells characterized by the effacement of microvilli and enfolded lymphocytes are present (Figure 1d1). Moreover, the presence of desmosomes between M-like cells and the neighboring cells reveals their enterocytic origin (Figure 1d1, inset). Using scanning electron microscopy analysis, we observed in mono-cultures that all Caco-2 cells possessed a regular brush border and well-developed tight junctions, whereas in co-cultures, approximately 20–30% of Caco-2 cells expressed short and irregular microvilli (Figure 1d2) [20]. Immunolabeling of M-like cells with CA19.9 and enterocytes with UEA-1 (Ulex europaeus isoagglutinin I) [21] indicated a similar percentage of conversion, assuming a surface equivalence for M and Caco-2 cells (Figure 1e1). This was further verified by co-localization of human IgA2 with M cells labeled with CA19.9 mAb (Figure 1e2), in agreement with Mantis et al. [4].

To verify functional Caco-2 cell conversion into M cells, the transport of yellow/green-conjugated, 0.2 µm nanoparticles (NPs) across mono- and co-cultures was examined. NPs have previously been used to study transcytosis in various M-like cell models *in vitro* and *in vivo* [22]. The number of transported NPs recovered in the basal medium was 5.5-fold higher in the co-cultures, compared to Caco-2 cell mono-cultures (*p*<0.001) (Figure 1f). The sum of these data confirmed that the *in vitro* model of human FAE allowed efficient Caco-2 cells to M-like cell conversion to occur (20–30%), and importantly, with a high level of reproducibility.

### Characterization of the Antibody Constructs

**Figure 2:**
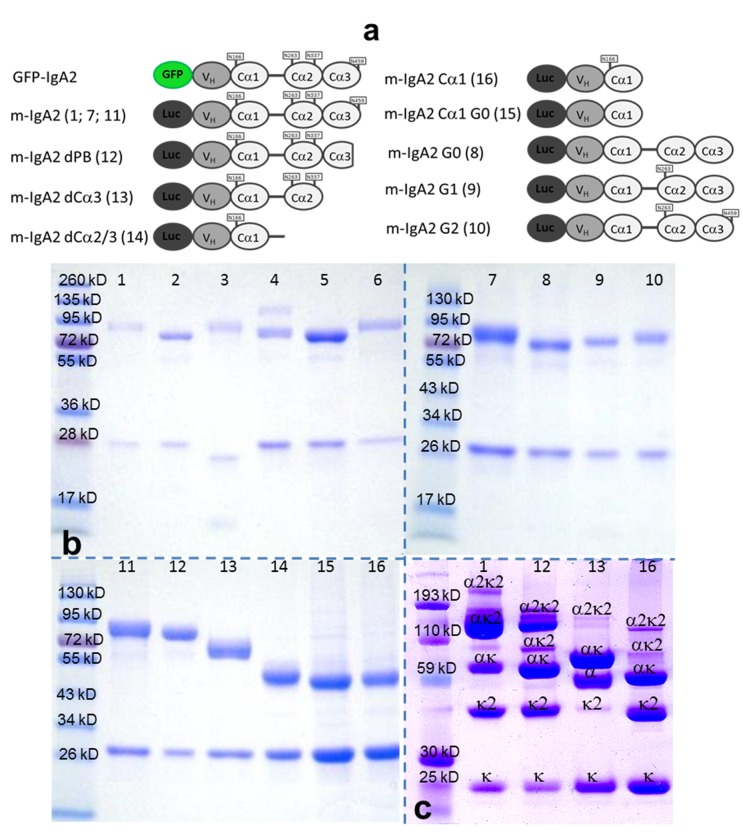
Antibody production. (a) Schematic representation of recombinant IgA2 constructs bearing GFP or Luciferase (Luc) at the N terminus. Numbers in brackets correspond to the lanes of gels depicted in (b) and (c). Glycosylation sites on asparagine residues (N) are indicated in rectangles connected to alpha chain domains. (b) Recombinant Igs separated by SDS-PAGE under reducing conditions and stained with Coomassie blue. (c) Analysis of a selection of recombinant Igs by SDS-PAGE under nonreducing conditions and stained with Coomassie blue. The identification of the various assembled forms was determined based on immunodetection with Abs specific to the alpha and kappa chains, respectively (not shown). 1, human m-IgA2 (monomer); 2, human d-IgA2 (dimer carrying the J chain); 3, murine m-IgA (monomer); 4, human IgE; 5, human IgG; 6, human m-IgA1 (monomer); 7, human mI-gA2 (monomer); 8, human m-IgA2 G0 (no glycosylation – monomer); 9, human m-IgA2 G1 (1 glycosylation site (Asn^263^) – monomer); 10, human m-IgA2 G2 (2 glycosylations sites (Asn^263^ and Asn^469^) – monomer); 11, human m-IgA2 (monomer); 12, human m-IgA2 dPB (monomer lacking the basal part); 13, human m-IgA2 dCα3 (monomer lacking the basal part and Cα3); 14, human m-IgA2 dCα2/3 (monomer lacking the basal part, and domains Cα3 and Cα2); 15, human m-IgA2 Cα1 G0 (Cα1 domain only, no glycosylation); 16, human m-IgA2 Cα1 (Cα1domain only).

Wt and truncated/mutated Ab constructs depicted in Figure 2 were cloned in the pGTRIO expression vector, stably transfected in CHO cells, produced in the culture supernatant, and purified by affinity chromatography as described in the Methods section. SDS-PAGE performed under reducing and nonreducing conditions confirmed the expected molecular weight for the light and heavy chains of the various constructs produced, and indicated assembly despite reduced formation of disulfide bridges between heavy and light chains (Figure 2), a feature commonly encountered while expressing IgA Abs in CHO cells [23].

### Structural Features Involved in the Specific Uptake and Transport of IgA2 in the *in vitro* Model of Human FAE

**Figure 3:**
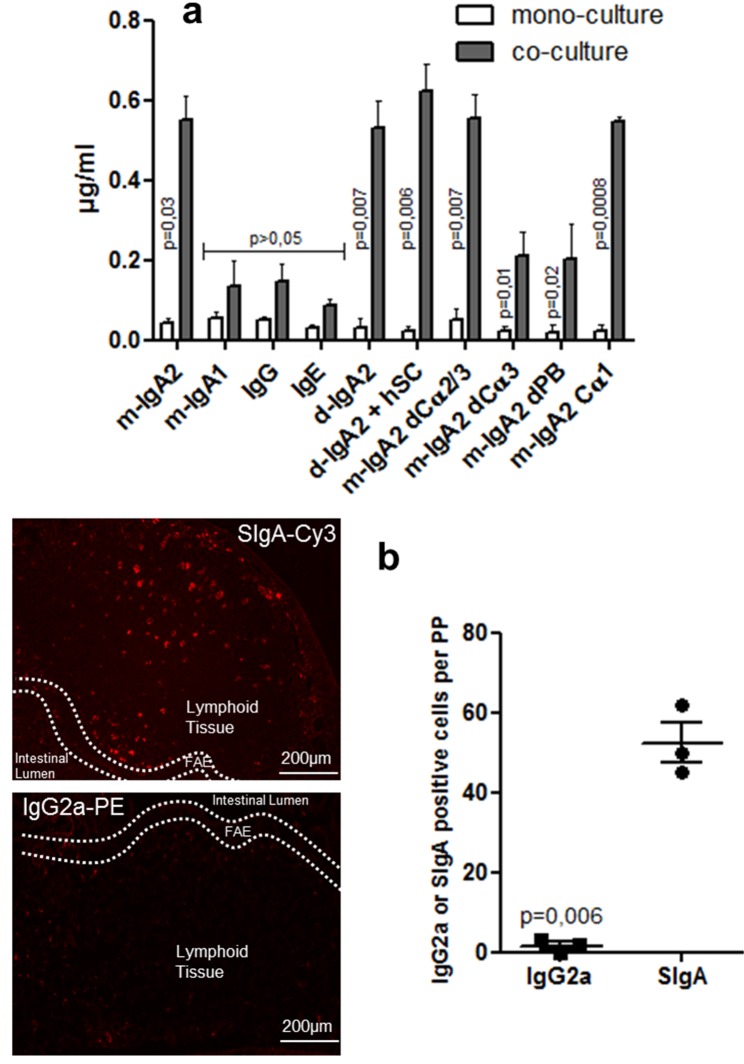
Specific transport of IgA2-Luc across the *in vitro* model of human FAE and in a mouse ligated loop containing a PP. (a) Mono- and co-cultures were incubated for 90 min at 37°C with various IgA2 constructs, and transported IgA was evaluated by associated luminescence (*n* = 4). The transport of m-IgA2, d-IgA2, d-IgA2+SC, m-IgA2 Ca1, and m-IgA2 dCα2/3 was significantly promoted in the co-cultures compared with the mono-cultures. (b) Only murine SIgA-Cy3, but not IgG2a-PE, incubated for 60 min in a mouse ligated intestinal loop is efficiently transported in underlying lymphoid tissues. Dotted lines delineate the interface between the intestinal lumen and tissue (FAE, side view). SIgA or IgG2a positive cells were quantified from three individual PPs.

One feature of M cells is their ability to transport a broad range of materials including Abs from the lumen to the underlying follicles. Specific retro-transport of Abs was compared between mono- and co-cultures using a luciferase (Luc)-IgA fusion protein. The Luc tag did not affect the Ab functionality (unpublished data) and allowed for sensitive quantification. As shown in Figure 3a, a significant exclusive transport of the IgA2 monomer (m-IgA2) across the cell monolayer harboring M-like cells was observed (*p* = 0.03). No significant transport of m-IgA1, IgG, or IgE was detected. Specificity of IgA reverse transcytosis was further confirmed *in vivo* by using a ligated murine intestinal loop. IgA positive cells were 30 times more abundant than IgG positive cells in PPs (Figure 3b). Dimerization, by incorporation of the J chain, or association with human SC did not modify IgA2 uptake by M-like cells (Figure 3a).

Next, mapping of regions and domains involved in IgA2 reverse transcytosis was performed with recombinant IgA2 lacking various portions of the heavy chain C-terminus (Figure 3a). IgA2 monomer depleted of Cα2, Cα3, and the tailpiece (m-IgA2 dCα2/3) crossed M-like cells as well as m-IgA2 wt, whereas IgA2 monomer depleted of the tailpiece (m-IgA2 dPB) and IgA2 monomer depleted of both the Cα3 and tailpiece (m-IgA2 dCα3) were not transported. The hinge region did not influence uptake as m-IgA2 dCα2/3 and m-IgA2 Cα1 (IgA2 with only the Cα1 constant region) gave similar results. Strikingly, M-like cell-mediated transport of IgA2 with only the Cα1 constant region was equivalent to wt m-IgA2. These results demonstrate that in the *in vitro* model, the Cα1 region of IgA2 is sufficient to allow reverse transcytosis through M-like cells.

### Influence of Glycosylation on the Uptake of IgA2 by M-Like Cells

**Figure 4:**
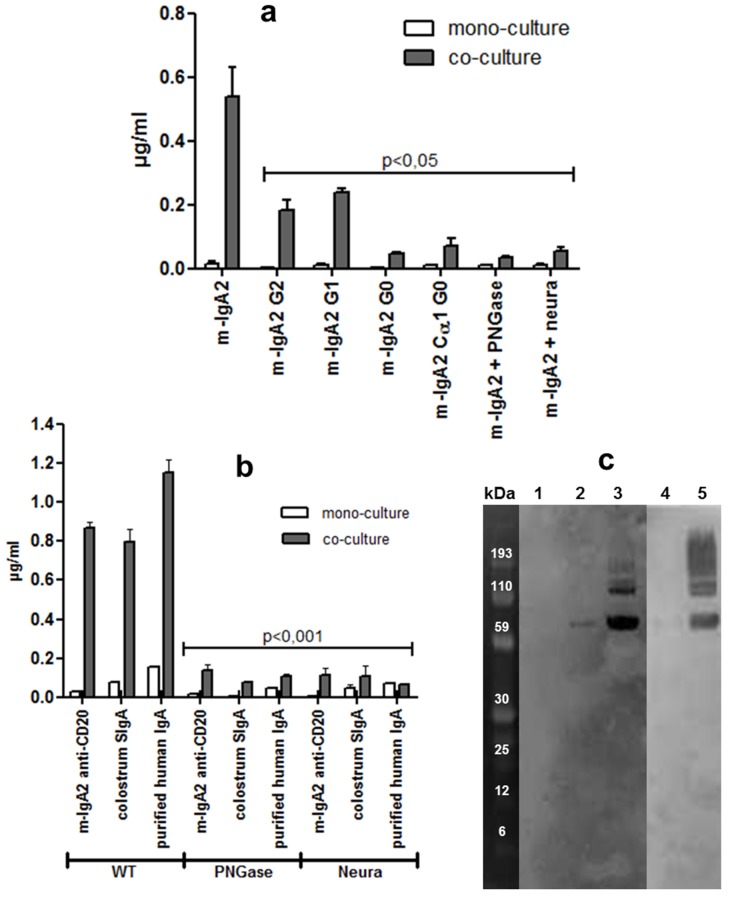
Influence of glycosylation and sialylation on the uptake of m-IgA2 by M-like cells. (a) Mono- and co-cultures were incubated for 90 min at 37°C with various hypoglycosylated, or enzymatically deglycosylated, IgA2 constructs, and transported recombinant IgA (m-IgA2) was evaluated by associated luminescence (*n* = 4). PNGase, Peptide N:glycosidase; Neura, neuraminidase. (b) Same set of experiments performed with recombinant anti-CD20 m-IgA2, human colostrum SIgA, and plasma-purified IgA; transported IgA was measured by ELISA. (c) IgA deglycosylation and desialylation was monitored by Western blot analysis using detection with HRP-conjugated lectins Ulex europaeus-1 and wheat germ agglutinin, respectively. Lane content: 1, m-IgA2 G0; 2, m-IgA2 G2; 3, m-IgA2; 4, colostrum SIgA+PNGase; 5, colostrum SIgA.

Subclasses of human IgA are also different with respect to the number of N-glycosylation sites. In order to determine whether N-glycans present on IgA2 could influence their uptake and transport by M-like cells, transcytosis of a battery of constructs with engineered glycosylation sites was compared. As shown in Figure 4a, the efficiency of reverse transcytosis was highly dependent on the number of glycosylation sites. Indeed, there was a significant decrease in the transport of m-IgA2 G2, G1, G0, and m-IgA2 Cα1 G0 compared with m-IgA2. These findings were confirmed by enzymatic digestion of m-IgA2 by PNGase, an amidase that cleaves between the innermost GlcNAc and asparagine residues of high mannose, hybrid, and complex oligosaccharides from N-linked glycoproteins.

Sialic acid (Sia) can occur in different glycosidic linkages, most typically at the exposed, nonreduced ends of oligosaccharide chains attached to a wide variety of proteins like IgA [24]. To assess the function of Sia in IgA binding to M-like cells, IgA was exposed to neuraminidase, which has the capacity to selectively cleave the glycosidic linkages of neuraminic acids. An important and significant decrease in transport of IgA2 lacking Sia, resembling that measured for m-IgA2 G0 or m-IgA2+PNGase, was observed. Identical results were obtained with another recombinant IgA2 Ab molecule specific for CD20, with SIgA purified from colostrum and with plasma IgA treated by neuraminidase or PNGase (Figure 4b). The absence of remaining carbohydrates or Sia on the different IgA was verified by Western blot using labeling with lectins (Figure 4c), while the integrity of the IgA2 polypeptide following enzymatic treatment was verified by analysis on SDS-PAA gels (unpublished data). These results demonstrate the essential role of IgA glycosylation sites, and in particular, Sia in the reverse transcytosis of IgA2 by M-like cells.

### IgA Binds to Intestinal M-Like Cells Via Dectin-1 and Siglec-5 Receptors

**Figure 5:**
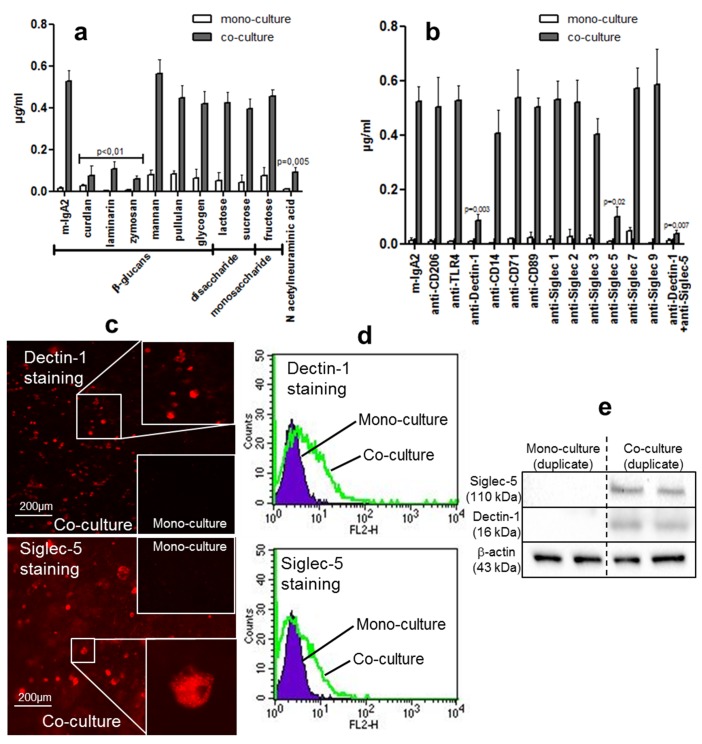
Structural mapping of sugars affecting transport of IgA2. (a) Mono- and co-cultures were pre-incubated apically with 5 mg of different mono/polysaccharides and sialic acid, prior to addition of various m-IgA2 constructs for 90 min at 37°C. The concentration of transported IgA was evaluated by associated luminescence (*n* = 4). (b) Similar experiments performed with 10 µg of blocking Abs directed against potential receptors (*n* = 4). Detection of Dectin-1 and Siglec-5 on M-like cells *in vitro* assessed by (c) immunofluorescence (top view), (d) flow cytometry, and (e) Western blot analysis using specific mAbs. Only cells or lysates recovered from co-cultures stained positive.

As the above results provide solid evidence of the contribution of glycosylation to reverse transcytosis, we postulated that the IgA2 receptor of M-like cells is a glucan receptor. Blocking experiments were performed using a series of β-glucans, mono-, and disaccharides. A statistically significant decrease in IgA2 transport was observed in the presence of β-glucans including curdlan, laminarin, and zymosan (Figure 5a). No inhibition was observed with other members of the family or with mono- or disaccharides.

To further explore the possible involvement of glycans in IgA2 binding to M-like cells, Abs directed against the most common sugar receptors were used in blocking experiments. The use of an anti-Dectin-1 mAb targeting this β-glucan receptor led to an almost complete inhibition of IgA2 reverse transcytosis (Figure 5b). In contrast, blocking of the mannose receptor with an anti-CD206 mAb or of the lipopolysaccharide receptor with anti-TLR4 and anti-CD14 mAbs did not influence IgA2 transport. Consistently, the presence of Dectin-1 was observed on M-like cells present in co-culture conditions only (Figure 5c–e). Other receptors that have been described as being involved in IgA transport were evaluated. Transferrin receptor expressed by enterocytes (CD71) [25], which binds IgA1 Abs, did not block IgA2 passage, thus confirming the exclusive transport of IgA2 by M-like cells. Similarly, targeting of the human myeloid IgA Fc receptor (CD89) [26] with a specific mAb did not block the transport of IgA2 (Figure 5b).

**Figure 6:**
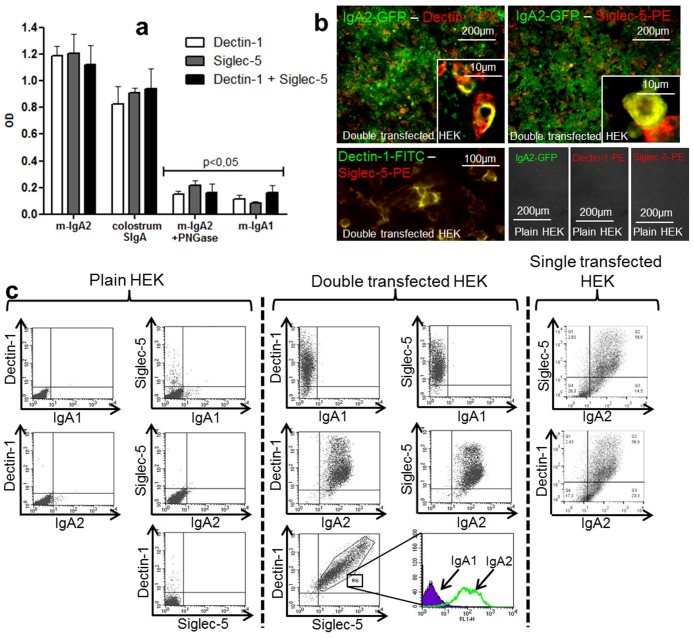
IgA2 interacts with Dectin-1 and Siglec-5–expressing cells. (a) Recognition by m-IgA2 of recombinant Dectin-1 and Siglec-5 used as coating molecules (*n* = 6), as measured by ELISA. (b) Determination by immunofluorescence of the binding of m-IgA2 to Dectin-1 and Siglec-5 receptors expressed by double-transfected HEK cells. Co-localization resulted in the appearance of yellow areas in the cell periphery. In control experiments, no binding of IgA1 was detected, and nontransfected HEK cells did not stain (*n* = 2). (c) In flow cytometry analysis, cells were plotted according to the FSC and SSC profiles and gated to include only HEK cells. A second selection was performed to include only those cells positive for Dectin-1 and Siglec-5.

To confirm the inability of desialylated IgA2 to target M-like cells *in vitro*, blocking experiments were also carried out using mAbs directed against various Siglecs, a family of receptors that specifically recognize Sia [27]. The unique involvement of Siglec-5 in IgA2 reverse transcytosis was demonstrated (Figure 5b), in contrast to all the other members of the family. The surface of M-like cells was Siglec-5^+^ in co-culture conditions only, with no labeling observed in mono-cultures (Figure 5c–e). Moreover, N-Acetylneuraminic acid severely affected SIgA2 reverse transcytosis (Figure 5a). Mabs to either Dectin-1 or Siglec-5 strongly inhibited transport of IgA2 *in vitro*, reaching up to 90% when added together (Figure 5b). Binding of monomeric IgA2 and SIgA to Dectin-1 and Siglec-5 was verified by ELISA using recombinant Dectin-1 and Siglec-5 as coating molecules (Figure 6a). In support of previous data, deglycosylated IgA2 and IgA1 were unable to recognize Dectin-1 and Siglec-5. Specificity of IgA2 recognition was further confirmed by immunofluorescence (Figure 6b) and flow cytometry (Figure 6c) using HEK cell transfectants expressing both Dectin-1 and Siglec-5. Importantly, co-localization between partners of the triad was observed in both types of analyses. Taken together, these results highlight the prominent role of Dectin-1 and Siglec-5 as receptors that mediate intestinal IgA2 reverse transcytosis.

### *In Vivo* Specific Uptake and Transport of SIgA Across the Murine and Human FAE

**Figure 7:**
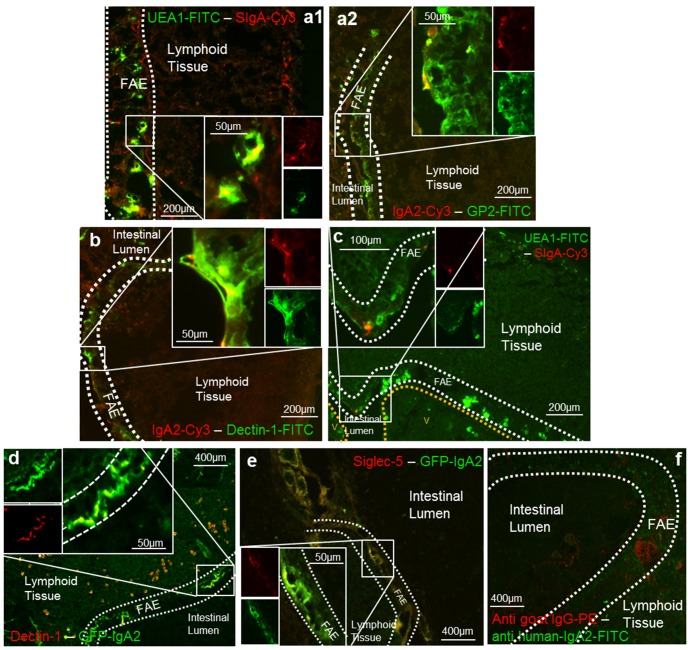
Specific uptake and transport of SIgA across murine and human FAE. (a) Mouse SIgA-Cy3 incubated for 60 min in a ligated intestinal loop containing a PP was taken up by M cells. Co-localization was observed with both UEA-1-FITC (a1) and GP2-FITC (a2) (*n* = 3). (b) Colocalization of SIgA-Cy3 with the Dectin-1 receptor stained in green with a specific mAb in the FAE overlying the PP (*n* = 2). (c) No entry of SIgA-Cy3 occurred in Dectin-1 KO mice (*n* = 2). (d–f) Images obtained from patient biopsy samples taken from the distal duodenum. V, villi. Biopsies were immunolabeled with human GFP-IgA2 and Dectin-1-PE (d) or Siglec-5-PE (e) at room temperature for 2 h. Conspicuous co-localization between GFP-IgA2 and Dectin-1-PE or Siglec-5-PE was observed (*n* = 2). (f) Negative controls were stained with secondary Abs alone (*n* = 2). On all pictures, dotted lines delineate the FAE separating the intestinal lumen and the lymphoid tissue (side view).

To verify the validity of data obtained using the *in vitro* model of human FAE, SIgA transport was also analyzed *in vivo* in a mouse ligated intestinal loop containing a PP [28]. As shown in Figure 7a1 and 7a2, mouse SIgA-Cy3 was present on the surface of, and inside, UEA-1^+^ or GP2^+^ M cells, thus confirming the *in vitro* binding data in the *in vivo* context. Co-localization of mouse SIgA on Dectin-1^+^ cells in the FAE confirmed the role of Dectin-1 in SIgA binding *in vivo* as well (Figure 7b). In support of these data, in a Dectin-1 KO mouse model, we observed no co-localization between SIgA-Cy3 and UEA-1^+^ M cells and no reverse transcytosis of SIgA-Cy3 in PPs (Figure 7c). The interaction between Dectin-1 or Siglec-5 with IgA2 was similarly observed in human PPs. Immunolabeling with green-labeled IgA2 and red-labeled Dectin-1 or Siglec-5 of patient biopsies displayed specific co-localization between the Ab and Dectin-1 (Figure 7d) or Siglec-5 (Figure 7e). No specific immunofluorescence of secondary IgG Abs was obtained on human M cells (Figure 7f).

### Mouse Oral Immunization with SIgA Serving as a Delivery Vehicle for HIV-1 p24

**Figure 8:**
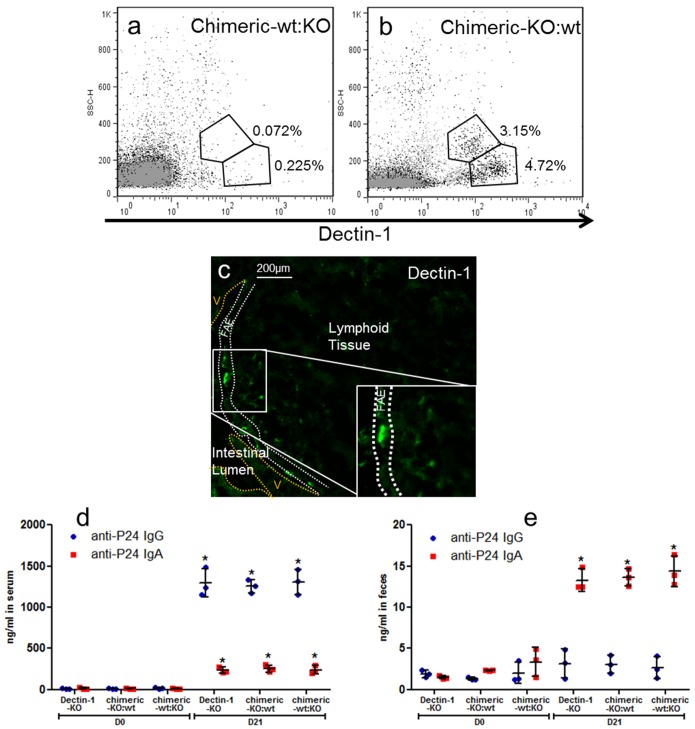
Phenotypic control of reconstituted chimeric mice and control subcutaneous immunization. Representative flow cytometry plots of peripheral blood leukocytes showing the absence of Dectin-1 expression by cells isolated from chimeric-wt:KO mice (a) and the presence of Dectin-1 on cells recovered from chimeric-KO:wt mice (b). Grey, control isotype; black, anti-Dectin-1 mAb. Cells were plotted according to FSC and SSC profiles and gated to include peripheral blood leukocytes. (c) Detection of Dectin-1 on the FAE of chimeric-wt:KO mice, as confirmed by immunofluorescence. V, villi. Subcutaneous immunization of Dectin-1 KO mice (*n* = 3), chimeric-KO:wt mice (*n* = 3), and chimeric-wt:KO mice (*n* = 3) using polylactoglycolic acid (PLA) nanoparticles as a delivery system for the HIV-1 p24 capsid protein. Ab production in serum (d) and feces (e) samples collected before immunization (D0) and 1 wk after the last immunization (D21) was examined by ELISA. Horizontal bars show the mean value ± SEM. Increase of IgG and IgA responses was observed in the serum and feces of all mice at D21 (*p<0.05).

These results prompted us to compare the outcome of oral immunization in wt C57BL/6 mice and Dectin-1 KO mice using SIgA as an intestinal delivery system targeting M cells. As Dectin-1 is also expressed by DCs or macrophages, one can argue that such cells intercalating within the FAE may “pollute” the Dectin-1 signal on M cells *in vivo*. To solve this issue, Dectin-1 KO mice reconstituted with wt bone marrow cells (chimeric-KO:wt) and wt mice reconstituted with Dectin-1 KO bone marrow cells (chimeric-wt:KO) were immunized. Confirmation of the correct reconstitution in the chimeric mice was obtained by flow cytometry (Figure 8a/b) and immunofluorescence (Figure 8c) analysis on peripheral blood leukocytes. Positive control of immunization was obtained by subcutaneous administration of nanoparticulated vaccine polylactic acid (PLA)-p24, which induced strong immune response in mice (Figure 8d/e) [29].

**Figure 9:**
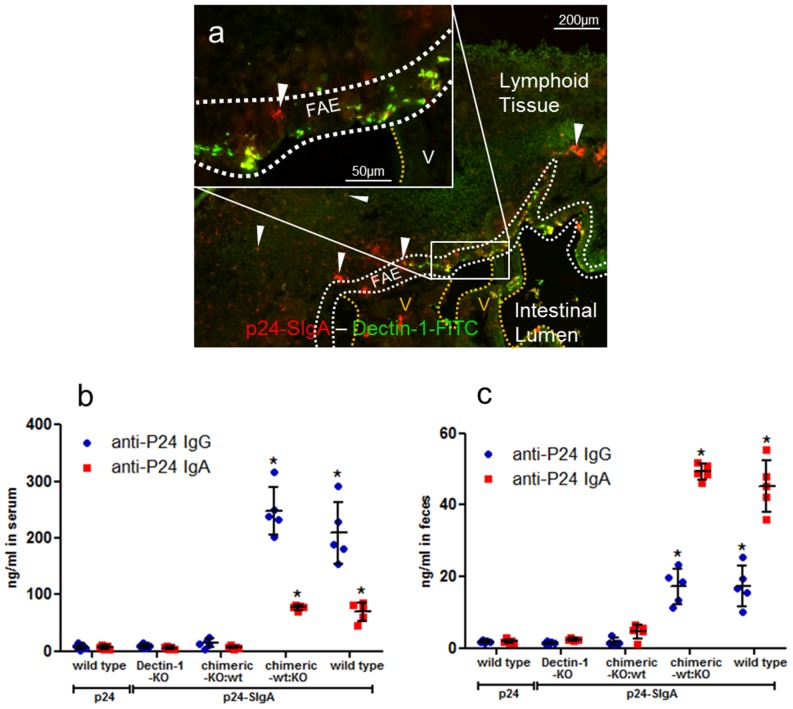
Dectin-1 on M cells is essential for SIgA uptake and elicitation of Ab responses against an associated Ag. (a) Incubation of p24-SIgA (red, indicated by arrowheads) for 90 min in a ligated intestinal loop containing a PP resulted in the targeting of the complex to Dectin-1^+^ M cells in the FAE, as indicated by the appearance of numerous yellow spots. V, villi. (b) Oral immunization of wt mice and chimeric-wt:KO (wt mice reconstituted with bone marrow cells from Dectin-1 KO mice) with HIVp24-SIgA resulted in the production of antigen-specific seric Abs, whereas Dectin-1 KO mice and chimeric-KO:wt mice (Dectin-1 KO mice reconstituted with bone marrow cells from wt mice) did not respond. (c) An identical pattern of Ag-specific Ab production was observed in the feces of immunized mice. Samples were collected 1 wk after the last immunization and Ab production was measured by ELISA. Horizontal bars show the mean value ± SEM (*p<0.05).

HIVp24 was chosen as a vaccine candidate antigen for its relatively low molecular weight, thus reducing the risk of disturbing the overall structure of SIgA after covalent coupling. Administration of p24-SIgA in an intestinal ligated loop resulted in the presence of the complex in the SED region of PPs (unpublished data). Moreover, p24-SIgA complexes administered orally co-localized with Dectin-1^+^ cells in the FAE region (Figure 9a). Oral immunizations with p24-SIgA were performed in wt, Dectin-1 KO, chimeric-wt:KO, and chimeric-KO:wt mice as described in the Methods section. As intestinal immunization is well known to induce both mucosal and systemic responses [30], serum and feces samples were collected 1 wk after the last immunization. p24-specific IgG and IgA titers were measured following immunization of wt and chimeric-wt:KO mice only (Figure 9b and 9c). Moreover, the levels of p24-specific IgG and IgA responses in these mice were 25-fold higher than those obtained after oral immunization with the p24 polypeptide only. No antigen-specific response was measured in Dectin-1 KO and chimeric-KO:wt mice, thus confirming the essential role of Dectin-1 in SIgA reverse transcytosis.

Taken together, these results indicate that reverse transcytosis of the p24-SIgA complex is strictly Dectin-1-dependent and results in the potentiated passage of the hooked Ag, which is subsequently processed to trigger the onset of mucosal and systemic Ab responses.

### Transcytosed SIgA Target DC-Specific Receptors *in Vivo* and *in Vitro*

**Figure 10:**
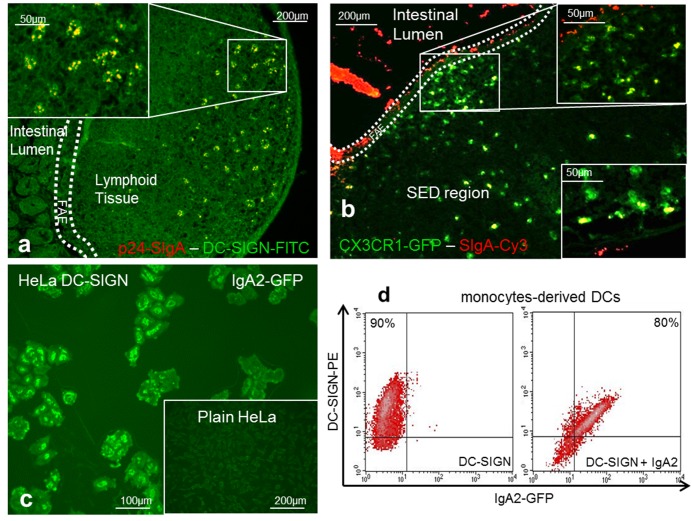
Transcytosed SIgA is taken up by DCs via the DC-SIGN receptor. (a, b) p24-SIgA or SIgA-Cy3 incubated for 60 min in a mouse ligated intestinal loop containing a PP were taken up respectively by DC-SIGN^+^DCs (a) and CX3CR1^+^DCs (b) in the SED region, as shown by co-localization in immunofluorescence images. (c) HeLa cells expressing DC-SIGN bound GFP-IgA2 having previously crossed the monolayer containing M-like cells *in vitro*. (d) Binding of IgA2 on DC-SIGN was detected by flow cytometry using monocyte-derived DCs. Cells were labeled with a specific mAb to DC-SIGN only (left panel), and in combination with IgA2-GFP (right panel). All experiments/analyses were carried out in at least duplicates.

In order to examine SIgA2 transport from the intestinal lumen to DCs located in the SED region of PPs, we took advantage of the recent demonstration that SIgA is recognized by DCs via the DC-SIGN receptor [31]. SIgA uptake by DCs was analyzed *in vivo* in a PP-containing ligated intestinal loop from wt mice with DC-SIGN-specific immunostaining and from CX3CR1-GFP transgenic mice. Figure 10a and 10b show specific localizations of p24-SIgA on DC-SIGN^+^ and SIgA-Cy3 on CX3CR1-GFP^+^ DCs present in the SED region. However, the strictly equivalent of DC-SIGN has not been described in mice, yet several homologues have been documented [32]. We assume that DC-SIGN-positive staining results from cross-reactivity with one of these murine homologues.

We next assess the relevance of these findings in the human in vitro system. HeLa transfectants stably expressing DC-SIGN added to the compartment bathing the basolateral pole of Caco-2 cells were used as surrogates of DCs populating the SED region of PPs [33]. Control of DC-SIGN expression was demonstrated by the inhibition of gp120 binding on HeLa-DC-SIGN^+^ cells by specific blocking mAbs (unpublished data). The binding of IgA2 that had previously crossed the monolayer containing M-like cells was observed by immunostaining of HeLa-DC-SIGN (Figure 10c), but not with wt HeLa cells used as a negative control. In another control, CHO expressing Langerin placed in the basolateral compartment did not bind transcytosed IgA2 (unpublished data). The transport of integral IgA2 through M cells, and also their preserved capacity to interact with DC-SIGN^+^ DC–expressing cells is another indication of the steps involved to ultimately lead to immune responses as detected above. These findings have also been confirmed by flow cytometry using human monocyte-derived DCs known to express DC-SIGN (Figure 10d).

The sum of these data shed light on the biochemical partners involved in reverse transcytosis of SIgA by PPs. SIgA is first taken up by M cells via the Dectin-1 receptor and/or Siglec-5, and is subsequently targeted to mucosal CX3CR1^+^ DCs bearing the DC-SIGN receptor. In the context of immune complexes, this process explains the functional production of mucosal and systemic Ab responses to the associated antigen.

## Discussion

M cells possess a high transcytotic capacity, allowing a wide range of materials to be transported including particulate Ags, soluble macromolecules, and pathogens. They are delivered from the intestinal lumen to inductive sites of the mucosal immune system. M cells are also the primary route through which SIgA are delivered to the GALT. Corthésy *et al.* have previously shown that after selective interaction with M cells, SIgA are targeted to DCs located in the SED region of PP, resulting in limited mucosal and systemic immune responses against a non-self-associated protein Ag [34]. Selective adherence to the apical surface of M cells is a prerequisite for efficient transepithelial transport, but the identity of receptors involved in SIgA endocytosis has remained elusive. In the current study, we investigated the transport of human SIgA2 across a model mimicking human FAE. At the level of IgA2, we provide evidence that both the Cα1 domain and associated glycosylation, more particularly Sia residues, are involved in M-like cell-mediated reverse transcytosis, while at the receptor level, both Dectin-1 and Siglec-5 have been identified as essential partner in the process. Finally, we validate our *in vitro* results upon analysis of murine and human tissues, ultimately demonstrating that Dectin-1/Siglec-5-mediated uptake of SIgA-based complexes results in productive mucosal and systemic antigen-specific Ab responses.

Initially, we studied reverse transcytosis of IgA2 across human M-like cells using a cell culture model that reproduces features of the FAE tissue. We confirmed that human IgA2, with or without J chain and/or bound SC, but not IgA1, IgG, or IgE, selectively bound to the apical surface of *in vitro* differentiated human M-like cells. Using a battery of deletion mutants, we demonstrated that domains Cα2 and Cα3 of IgA2 are dispensable to keep reverse transcytosis through M-like cells highly active. Low or absent transport of m-IgA2 dCα3 and m-IgA2 dPB comprising the Cα1 region suggests that subtle structural changes may affect optimal folding of these two particular recombinant proteins. Our *in vitro* results obtained with human cells do not totally correlate with the *in vivo* results of Mantis et al., who showed that both domains Cα1 and Cα2 were required for IgA binding to mouse PP M cells [4]. Differences in the expression systems for IgA constructs (deletion versus domain swab) and the glycosylation pattern may explain this discrepancy. It is conceivable that a critical density of glycans must be present to ensure uptake, as was recently described for Dectin-1 efficiently binding β-glucan polymers [35]. In conclusion, our study unequivocally demonstrates that IgA transport requires the presence of the properly glycosylated Cα1 domain within the Ab structure. The model opens the path toward *in vitro* assays of transport across reconstituted FAE, examination of the mechanisms of uptake, and investigation into vaccine or intestinal microbe delivery.

Sia residues on pathogens interact with Siglecs, which are expressed in the hemopoietic, immune, and nervous systems. Glycosylation patterns on pathogens are frequently used for adherence to, and passage across, the mucosal epithelium and in particular M cells in the FAE [36]–[38]. Similarly, it is conceivable that abundant carbohydrates located on the surface of SIgA may intervene in the process of selective recognition of M cells. The sum of our data confirms this working hypothesis, and demonstrates the prominent influence of glycosylation on the uptake of IgA2 by M cells. Additional experiments dealing with deletion of particular glycosylation sites and enzymatic desialylation allowed us to confirm the role of Sia residues in reverse transcytosis (Figure 4). Several members of the β-glucan superfamily were also identified as competitors of IgA2 transcytosis (Figure 5). This adds to the multiple functions of carbohydrates in SIgA including, for example, neutralization of bacterial toxins [39] and interaction with commensal bacteria [40].

Having unraveled the structural features responsible for the selective transport of SIgA in the reconstituted FAE model, we sought to identity the receptor(s) by which SIgA is taken up and transported by M cells. The use of blocking Abs against known IgA receptors including CD89 and CD71 did not prevent SIgA2 reverse transcytosis. These data, combined with the sufficient role of the Cα1 region of IgA2 in M-cell-mediated reverse transcytosis, led to the conclusion that no other known IgA receptor (pIgR, Fcα/μ receptor, and the asialoglycoprotein receptor) was involved in the process. Given the established involvement of Sia and β-glucan moieties, we speculated that the IgA2 receptor of M cells is a glucan- and/or Sia-receptor. Our work provides evidence of the presence of Dectin-1 on M-like cells, together with its involvement in reverse transcytosis of SIgA2. Dectin-1 is a type II transmembrane protein of the C-type lectin family, expressed by myeloid phagocytes (macrophages, DCs and neutrophils), which recognizes β-glucans in fungal cell walls and transduces signals triggering phagocytosis and the production of reactive oxygen species [41],[42]. In contrast, as recognition of soluble ligands by Dectin-1 does not lead to inappropriate activation signaling [35], its presence on M cells is consistent with simple SIgA capture and internalization.

Co-operation between Fc galactosylation and Dectin-1–inducing anti-inflammatory activities suggests that Dectin-1 is capable of working in combination with other partners in the cell plasma membrane. In view of the involvement of Sia in IgA2 reverse transcytosis via M cells, we investigated whether a Siglec receptor could serve this function. The majority of Siglecs, including CD33-related Siglecs like Siglec-5, appears to be naturally masked owing to cis-interactions with adjacent Sia. Unmasking of Siglecs can also occur in some cases by cellular activation or by exposure to sialidases. The unmasked Siglec would then be capable of *de novo* interactions with surrounding ligands in the environment. This could result in increased interactions with exogenous materials including glycosylated SIgA. Such a scenario of Siglec serving as a co-receptor has been reported in the case of HIV-1 entry mediated by CD4 in macrophages.

Preparation of murine duodenal ligated loops validated the results generated in the *in vitro* model of human FAE. This method has proven valuable in documenting the interaction of mouse IgA with PP M cells [4]. Tissue immunolabeling both confirmed the transport of SIgA2 by UEA-1^+^ and GP2^+^ M cells, and that of murine SIgA by Dectin-1 (Figure 7a and b). However, the absence of cross-reactivity of the anti-human CD170 mAb prevented us from confirming the role of Siglec-5 in the reverse transcytosis of SIgA in mice. Consistent with the *in vitro* data gathered in the model based on human cells, human biopsy analyses resulted in specific co-localization between IgA2-GFP and Dectin-1 (Figure 7d) or Siglec-5 (Figure 7e).

Finally, oral immunization of wt, Dectin-1 KO, or chimeric mice with p24-SIgA complexes unambiguously demonstrated that reverse transcytosis of SIgA is strictly dependent on Dectin-1 expressed on M cells. The further confirmation of the essential role of Dectin-1 in the *in vivo* context provides an explanation to the uptake of antigen-bearing SIgA by M cells, a feature resulting in systemic and mucosal immune responses [43]. The lack of a murine functional ortholog of human Siglec-5 prevented us from confirming the associated role of Siglec-5 in SIgA reverse transcytosis *in vivo* [44]–[46].

*In vivo*, the uptake of murine SIgA by murine CX3CR1^+^ DCs present in the GALT could also be documented (Figure 10b). In the SED region, CX3CR1^+^ DCs play a central role in antigen sampling [47]. In contrast to CD103^+^ DCs, CX3CR1^+^ cells represent a nonmigratory gut-resident population, which displays poor T-cell stimulatory capacity [48],[49]. In contrast to CD103^+^ DCs that serve classical DC functions and initiate adaptive immune responses in local lymph nodes, CX3CR1^+^ populations might modulate immune responses directly in the mucosa and serve as a first line barrier against invading enteropathogens. This supports the low activation properties of SIgA targeting antigen to DCs in the SED region [50],[51]. A recent study has shown that small intestine goblet cells function as passages delivering the low molecular weight soluble dextran (10 kDa) to CD103^+^ DCs [52], which promote IgA production, imprint gut homing on lymphocytes, and induce the development of regulatory T cells. As HIVp24 is administered in the form of a complex with SIgA (400 kDa), we believe that this pathway need additional characterization before it can be considered as operative for large molecules.

Transcytosis across M cells is known to enable the selective transport of particulate antigens in the absence of any assessable damage [53],[54]. This holds true for soluble SIgA, as the transcytosed Ab released by M cells in the human *in vitro* and murine *in vivo* models was still able to specifically target cells expressing DC-SIGN in the basolateral environment (Figure 10a/c/d). In mucosal tissues such as the rectum, uterus, and cervix, DC-SIGN is abundantly expressed by DCs present in the lamina propria and PPs, further substantiating the importance of the localization of DC-SIGN^+^ DCs as a first line of defense against viruses and pathogens. Delivery in the form of SIgA-based immune complexes may thus combine the onset of limited immune responses, which translates into the absence of spurious inflammatory reactions. Moreover, this receptor, by binding to ICAM-3, favors the generation of antigen-specific suppressive CD4^+^ T cells, which produce IL-10 [55], a cytokine that intervenes in both intestinal homeostasis and the production of local IgA.

This work defines Dectin-1 expressed on the surface of M cells as a receptor involved in SIgA reverse transcytosis both *in vitro* and *in vivo*. Besides bringing new information on the mechanism involved in SIgA retro-transport, deciphering the identity of such receptors may lead to the further development of mucosal vaccines targeting M cells. In future work, it will be critical to test the expression of Dectin-1 on other mucous membranes such as nasal/bronchial, endocervical, or buccal mucosa order to evaluate the broad applicability of this finding to active and passive immunization. As a perspective to future works, one can argue that intestinal villous M cells serving as an antigen gateway for the sampling of gut bacteria and inducing Ag-specific immune responses in a PP-independent manner [56] may contribute to SIgA reverse transcytosis as well.

## Methods

### Reagents

Pullulan from *Aureobasidium pullulans*, mannan from *Saccharomyces cerevisiae*, α-Lactose, L-fructose, glycogen from bovine liver, sucrose, curdlan from *Alcaligenes faecalis*, laminarin from *Laminaria digitata*, and zymosan from *Saccharomyces cerevisiae* were all purchased from Sigma-Aldrich.

Anti-human Dectin-1/CLEC 7A polyclonal Ab (pAb) (goat IgG), anti-human CD14 mAb (mouse IgG1), anti-human TLR4 pAb (goat IgG), anti-human CD170 mAb (Siglec-5) (mouse IgG1), and anti-human CD329 mAb (Siglec-9) (mouse IgG2a) were all purchased from R&D Systems. Anti-human CD206 mAb (mouse IgG1) (mannose receptor) was purchased from Ozyme. Anti-human CD22 mAb (Siglec-2) (mouse IgG1), anti-human CD33 mAb (Siglec-3) (mouse IgG1), anti-human CDw328 mAb (Siglec-7) (mouse IgG1), and anti-human CD169 mAb (Siglec-1) (mouse IgG1) were purchased from AbD Serotec. Anti-human CD71 mAb (mouse IgG1) was purchased from Cliniscience. Anti-human CD89 mAb (mouse IgG1) was purchased from Abcam. All Abs were blocking and used according to the procedure provided by the manufacturer.

Yellow-green carboxylated or aminated latex particles (FluoSpheres) with a mean diameter of 0.2 µm were purchased from Molecular Probes.

### Cell Culture

Both the human intestinal cell line Caco-2 cell (clone 1) (obtained from Dr. Maria Rescigno, University of Milan-Bicocca, Milan, Italy) [57] and CHO cells were cultured in Dulbecco's modified Eagle's medium (DMEM) (PAA) supplemented with 10% (v/v) fetal bovine serum (FBS, Thermo-Fisher), 1% (v/v) nonessential amino-acids (PAA), and 1% (v/v) penicillin-streptomycin (PAA). The human Burkitt's lymphoma cell line Raji B (American Type Culture Collection) was cultured in RPMI 1640 supplemented with 10% (v/v) FBS, 1% (v/v) nonessential amino-acids, 1% (v/v) L-glutamine, and 1% (v/v) penicillin-streptomycin.

### Inverted *in Vitro* Model of the Human FAE

The inverted FAE model (Figure 1a) has been previously reported [20]. Several major changes were made and are listed below. Inverted Transwell polycarbonate inserts (12 wells, pore diameter of 3.0 µm, Corning) were coated with Matrigel, a basement membrane matrix (BD Biosciences) prepared in pure DMEM to a final protein concentration of 100 µg/ml for 1 h at room temperature. The coating solution was removed and inverted inserts washed with 300 µl of DMEM. Caco-2 cells (3×10^5^), resuspended in 300 µl of supplemented DMEM, were seeded on the lower insert side and cultured overnight. The inserts were then inverted and placed in a 12-well culture dish and kept for 9 d. Raji B cells (5×10^5^), resuspended in supplemented DMEM, were then added to the basolateral compartment of the Caco-2 cells, and co-cultures were maintained for 5 d. Mono-cultures of Caco-2 cells, cultivated as above but without the Raji B cells, were used as controls. Finally, the inserts were inverted in six-well plates, and a piece of silicon tubing (14×20 mm, Labomoderne) was placed on the basolateral side of each insert. Cell monolayer integrity, both in mono- and co-cultures, was controlled by measurement of TEER using an Endohm tissue resistance chamber (Endohm-12, World Precision Instruments) connected to a Millicell-ERS Ohmmeter (Millipore). The resistance of medium alone (9 Ω×cm^2^) was considered as background resistance and subtracted from each TEER value. Barrier function of the tight junctions was also analyzed by zonula occludens-1 (ZO-1) immunolabeling (see next section).

### Establishment of the *in Vitro* Model of Human FAE

Cells morphologically similar to M cells were discriminated from Caco-2 cells using transmission electron microscopy (TEM) and scanning electron microscopy (SEM). TEM and SEM were used to evaluate morphological cell changes after co-culture with Raji cells. Mono- and co-cultures were washed twice in HBSS and fixed in 4% (v/v) formaldehyde. Ultra-thin sections of cell-covered filters were prepared for TEM analysis by standard methods, as previously described [58]. Observations were made using a Hitachi H-800 and a Digital camera Hamamatsu AMT XR40. Samples processed for SEM analyses were dehydrated, dried at critical point, and gold coated. Pictures of cell monolayers were obtained with a Thermo Noran Quest 2 L Hitachi S 3000N. Since no human-specific M cell markers have yet been identified, the microvilli-free morphology of M-like cells was used to identify and quantify them by SEM. Mono-cultures were used as controls.

Characterization and quantification of M-like cells in co-cultures was further verified by immunolabeling. Inserts were washed in HBSS to eliminate residual medium, incubated in 4% paraformaldehyde for 30 min, permeabilized with 0.1% Triton X-100 (Sigma-Aldrich), and blocked with PBS containing 5% FBS for 15 min at room temperature. Immunolabeling was performed using a combination of GFP-IgA2, anti-human ZO-1 mAb (Invitrogen), and mouse anti-human CA19.9 (Dako) [10]. Each reagent was diluted to 1/100, and incubated for 2 h at room temperature. 1/200 dilutions of secondary antibodies labeled with a fluorochrome were incubated for 1 h at room temperature. After two washes, inserts were air-dried, mounted with Fluoprep (BioMerieux), and observed by Immunofluorescence microscopy (Eclipse Ti, Nikon).

### Transport of Nanoparticles in the *in Vitro* Model

Nanoparticle (NP) (yellow-green fluorescent, 0.2 µm carboxylate-modified FluoSpheres beads) transport by polarized Caco-2 cells was evaluated in HBSS medium. NP concentration was adjusted to 4.5×10^9^ NPs/ml and vortexed for 1 min to dissociate possible aggregates. NP suspension was added to the apical side of cell monolayers (400 µl) and the inserts were incubated at 37°C for 90 min. Basolateral solutions were then sampled and the number of transported particles was measured by flow cytometry (Facs Calibur, Becton Dickinson). The measurements were based on both fluorescence and particle size.

### Antibody Production

Light and heavy chain encoding genes from a human TNF-alpha–specific IgA Ab were cloned in a single vector (pGTRIO) designed for efficient Ab expression in HEK293 and CHO cell lines. pGTRIO is a derivative of pVITRO2 (Cayla-InvivoGen, Toulouse, France), a multigenic plasmid that contains two distinct transcription units. In pGTRIO, the antibiotic resistance gene is under the control of the EF1 alpha/HTLV promoter combined with the CMV enhancer that together constitutes a third transcription unit with the EF1 polyadenylation signal. The kappa constant region was cloned downstream of the FerL promoter together with the CMV enhancer, and the heavy chain constant regions were cloned downstream of the FerH promoter together with the human aldolase A enhancer. Unique restriction sites were introduced upstream of each constant region in order to allow the cloning of the variable region as SgrAI-BsiWI and AgeI-NheI fragments for VL and VH, respectively. All variable heavy chain regions were fused at the C-terminal end of secreted luciferase. CHO cells were transfected with pGTRIO constructs using the LyoVec system (Cayla-InvivoGen) in accordance with the manufacturer's instructions. Stable transfectants were selected in antibiotic-containing medium and screened for the production of Abs with a neutralizing activity on the HEK-Blue TNF-alpha/IL1-beta reporter cells (Cayla-InvivoGen) stimulated with TNF-alpha. IgA preparations were purified using Kappa affinity chromatography, IgG preparations were purified using protein G affinity chromatography, and IgE preparations were purified using protein L affinity chromatography. Ig-Luc constructs specific for TNF-alpha maintained their ability to block the cytokine, indicating proper assembly and folding. The following Abs were obtained by this method (Figure 2): human m-IgA2 (monomer); human GFP-IgA2 (monomer); human d-IgA2 (+ J chain - dimer); murine m-IgA (monomer); human IgE; human IgG1; human m-IgA1 (monomer); human m-IgA2 G0 (no glycosylation – monomer); human m-IgA2 G1 (1 glycosylation (Asn^263^)- monomer); human m-IgA2 G2 (2 glycosylations (Asn^263^ and Asn^469^) – monomer); human m-IgA2 dPB (without basal part – monomer); human m-IgA2 dCα3 (without basal part and Cα3 – monomer); human m-IgA2 dCα2/3 (without basal part, Cα3 and Cα2 – monomer); human m-IgA2 Cα1 G0 (only Cα1 without glycosylation); and human m-IgA2 Cα1 (only Cα1 with glycosylation). M-IgA2 was desialylated and deglycosylated with neuraminidase and PNGase, respectively (Enzymatic CarboRelease Kit, QA-Bio). Purity and assembly of the Abs were controlled by SDS-PAGE (Figure 2b/c). IgA deglycosylation was detected under normal conditions by using standard Western blot protocol with a combination of UEA-1-HRP and WGA-HRP lectins (Sigma-Aldrich).

For visualization of the mouse SIgA retrotranscytosis, a polymeric IgA Ab from the hybridoma clone IgAC5 specific to *S. flexneri* serotype 5a LPS [59] was obtained as previously described [60]. Purified free human SC was produced in Chinese hamster ovary cells [61]. SIgA molecules were obtained by combining in PBS pIgA molecules with a 2-fold excess of human SC for 2 h at room temperature according to the conditions described in the study by Rindisbacher et al. [62]. Cy3-SIgA molecules were obtained by conjugation with indocarbocyanine (Cy3) using the FluoroLink mAb Cy3 labeling kit (Amersham Biosciences) according to the procedure provided by the manufacturer.

### Antibody Transport in the *in Vitro* Model

Transport experiments were performed in HBSS at 37°C for 90 min with 10 µg of Ab conjugated with luciferase (Luc). Basolateral solutions were then recovered and the number of retro-transcytosed Ab-Luc measured by luminometry (Tristar LB941, Berthold Technologies) using the Gaussia Luc Assay Kit (Biolux) according to the procedure provided by the manufacturer. Ab-Luc transport was expressed as a mean value ± S.E.M. For inhibition experiments, cell monolayers were first preincubated apically with 5 mg of inhibitor in HBSS for 90 min at 37°C, and washed with HBSS, before adding the Ab-Luc suspension. All transport experiments were carried out in triplicate and were standardized with m-IgA2 (ratio RLU/µg of Ab).

### Dectin-1 and Siglec-5 Specific ELISAs

Maxisorp 96-well plates were either coated with 50 µl of recombinant human Dectin-1/CLEC7A (5 µg/ml) (R&D Systems), 50 µl of recombinant human Siglec-5 (5 µg/ml) (R&D Systems), or 50 µl of an equal mixture of both Dectin-1 and Siglec-5 proteins and incubated O/N at 4°C. The wells were then washed three times with PBS and saturated with 200 µl of blocking solution (PBS+3% BSA) at room temperature for 1 h. The blocking solution was then discarded and 100 µl of m-IgA2, colostrum IgA, m-IgA2+PNGase, or m-IgA1 were added at a concentration of 5 µg/ml. After 1 h of incubation at room temperature, wells were washed three times with PBS, and bound IgA was detected using biotinylated goat anti-human IgA (Southern Biotech) followed by streptavidin-HRP (Amersham). Results are expressed as the means of OD ± SEM.

### Protein Administration Into Ligated Loops

Six-week-old C57BL/6 mice were purchased from Charles River Laboratories (Lyon, France). CX3CR1-GFP transgenic mice were obtained from Maryline Cossin (Joseph Fourier University, France). Dectin-1 knockout mice [63], chimeric-KO:wt mice, chimeric-wt:KO mice, C57BL/6 mice, and CX3CR1-GFP transgenic mice were hosted at the University Hospital Unit for animal testing (Saint-Etienne, France). For ileal loop preparation, mice were starved overnight, anesthetized by intra-peritoneal injection of a mix of ketamine and xylazine (100 and 10 mg/kg animal weight, respectively), and kept warm at 37°C throughout the surgical procedure. We administered 100 µl of a 1 mg/ml solution of SIgA-Cy3 or p24-SIgA diluted in PBS into a 1.5-cm ileal loop containing a PP. Upon completion of the experiment, the mice were sacrificed by cervical dislocation and the piece of intestine was removed, extensively washed with PBS, fixed for 2 h in 3% paraformaldehyde, and included in optimal cutting tissue (OCT) embedding solution. We captured 7-µm sections (Leica cryostat model CM1950, Leica Microsystems) on Ultra+ superfrost microscope slides (VWR International) and stained for M cells. Slides were washed in PBS to eliminate residual OCT embedding solution, and blocked with PBS containing 5% FBS for 30 min at room temperature. Abs diluted to 20 µg/ml were incubated for 2 h at room temperature. The slides were then washed in PBS, air-dried, and mounted with Fluoprep (Biomérieux). Slides were observed by immunofluorescence microscopy (Eclipse, Nikon). Immunolabeling was performed using a combination of UEA-1-FITC (Sigma-Aldrich), anti-human Dectin-1/CLEC 7A pAb, anti-human CD170 mAb (R&D Systems), anti-human GP2 mAb (MBL), and p24-specific Ab directly labeled with PE (Santa Cruz Biotechnology). The protocol followed the guidance of the regional Ethics Committee for Animal Testing (CREEA) (Permit Number No. 69387487).

### Immunolabeling of Human Peyer's Patches *ex Vivo*

Informed and consenting patients who had undergone upper duodenal endoscopy for routine diagnostic purposes (e.g., dyspepsia and chronic diarrhea) with normal intestinal mucosa provided four to six biopsy samples from the distal duodenum. Biopsies were fixed for 2 h in 3% paraformaldehyde and included in OCT embedding solution, before being cryosectioned using a Leica cryostat model CM1950. We captured 7 µm sections on Ultra+ Superfrost microscope slides, and they were stained for M cells as described for mouse intestine and observed by immunofluorescence microscopy. Immunolabeling was performed using a combination of GFP-IgA2, anti-human Dectin-1/CLEC 7A pAb, and anti-human CD170 mAb (R&D Systems).

### Generation of Dectin-1 Chimeric KO:wt and wt:KO Mice

We housed 5–9-wk-old Dectin-1 KO and wt C57BL/6 males in individually ventilated cages at least 7 d prior to being irradiated. During this time and throughout the remainder of the experiment, animals were also maintained on sterile food and acidified in sterile water (containing 0.004% HCl). Animals received two doses of full body irradiation at 5 Gy (2×500 rads). Each dose was separated by a 3 h interval to limit gastrointestinal problems. Irradiated mice were returned to individually ventilated cages for 24 h. Bone marrow was isolated from the femurs and tibia of donor Dectin-1 KO and wt C57BL/6 males under sterile conditions in the absence of red blood cell lysis. Nucleated cells were counted on a haemocytometer. Irradiated Dectin-1 KO mice each received 2×10^6^ total nucleated bone marrow cells from wt C57BL/6 mice intravenously via the lateral tail vein. The phenotype of these mice, named chimeric-KO:wt, is thus wt at the systemic level and Dectin-1 KO at the mucosal level. Irradiated wt C57BL/6 mice were similarly injected with the same number of Dectin-1 KO donor cells. The phenotype of these mice, referred to as chimeric-wt:KO, is Dectin-1 KO at the systemic level and wt at the local level. Animals were maintained in individually ventilated cages as described above for a further 6 wk. Five weeks after bone marrow injections, 50 µl of tail vein blood was taken from each animal to characterize the cell phenotype, and red blood cells lysed for 2 min at room temperature in 1× Pharmlyse buffer. Cells were then washed twice in phosphate-buffered saline and counted. Cells were incubated for 15 min in FACS block (HBSS+2 mM NaN_3_, 0.5% BSA, and 5% heat inactivated rabbit serum) containing 6 µg/ml Fc-receptor blocking mAb (clone 24G2), prior to addition of 10 µg/ml biotinylated anti-Dectin-1 mAb (clone 2A11) or the rat biotinylated isotype control IgG2b for 30 min on ice. After three washes in FACS wash (HBSS complemented with 2 mM NaN_3_ and 0.5% BSA), cells were incubated in FACS block containing 1/200 APC-conjugated streptavidin (Invitrogen) for 20 min on ice. Cells washed three times were analyzed on a FACSCalibur (Becton-Dickinson) and data analyzed using FlowJo software.

### *In Vivo* Delivery of HIVp24-SIgA and Measurement of p24-Specific Abs

#### Mice

Dectin-1 KO, chimeric-KO:wt, chimeric-wt:KO, and wt C57BL/6 mice (Charles River Laboratories, France) were hosted at the University Hospital Unit for animal testing (Saint-Etienne, France). The protocol followed the guidelines of the Ethics Committee of CREEA (Permit Number No. 69387487).

#### HIVp24-SIgA complexes

HIV-1 p24 capsid protein from clade B strain (Px Therapeutics, France) was covalently associated to polymeric SIgAC5 using the Sulfo-KMUS heterobifunctional crosslinker (Thermo Scientific). Covalent complex formation was verified by Western blot with a polyclonal anti-HIV-1 serum and revealed with anti-human IgG HRP-conjugated secondary Ab (Amersham).

#### Immunization with HIVp24-SIgA complexes

Mouse oral immunizations were performed by orogastric intubation with polyethylene tubing under light anesthesia with isofluroan (Halocarbon Laboratories). The tubing was introduced at a fixed distance of 1.8 cm from the incisors. Immunizations consisted of three administrations of 100 µl at 1-wk intervals. Dectin-1 KO (*n* = 5), chimeric-KO:wt (*n* = 5), and chimeric-wt:KO (*n* = 5) mice were immunized with 100 µg of HIVp24-SIgA per administration.

#### Immunization with HIVp24-PLA complexes (positive control group)

Dectin-1 KO (*n* = 5), chimeric-KO:wt (*n* = 5), chimeric-wt:KO (*n* = 5), and wt (*n* = 5) C57BL/6 mice were immunized with 10 µg of HIVp24-PLA nanoparticles (suspended in 100 µl of PBS) by subcutaneous route. Immunizations consisted of three administrations of 100 µl at 1-wk intervals.

#### Measurement of HIVp24-specific IgG and IgA Abs

Serum and feces samples were recovered 1 wk after the last immunization. Five fresh feces were collected from each animal. Feces were incubated with Halt Protease Inhibitor Cocktail (Thermo Scientific), centrifuged at 16,000× *g*, and stored at −20°C until use. Specific Abs against HIVp24 were measured using a quantitative ELISA. Maxisorp 96-well plates were coated with either 50 µl of HIVp24 Ag solution (5 µg/ml in sterile PBS) or 50 µl of a 1/3,200 dilution of an equal mixture of anti-mouse Ig kappa and lambda light chain–specific mAbs (Serotec), and then incubated O/N at 4°C. Murine IgG or IgA immunoglobulins (Igs) (Southern Biotech) were used as standards. Bound or captured Igs were detected by incubation with HRP-conjugated goat anti-mouse (IgG), while IgA was detected using biotinylated goat anti-mouse IgA (Southern Biotech) followed by streptavidin-HRP (Amersham). Results are given as the means of concentrations ± SEM.

### Statistical Analysis

Statistical analyses were performed using the InStat version 2.01 from the GraphPad Software, and the unpaired two-tail Mann–Whitney U test was applied. Significance limit was set at *p*≤0.05.

## Abbreviations

FAE: follicle-associated epithelium
GALT: gut-associated lymphoid tissue
Luc: luciferase
M: Microfold
NPs: nanoparticles
PLA: polylactic acid
PP: Peyer's patches
SEM: scanning electron microscopy
Sia: Sialic acid
SIgA: secretory IgA
TEER: transepithelial electrical resistance
TEM: transmission electron microscopy
UEA-1: Ulex europaeus agglutinin–1
